# Giant Polymersome Protocells Dock with Virus Particle Mimics via Multivalent Glycan-Lectin Interactions

**DOI:** 10.1038/srep32414

**Published:** 2016-08-31

**Authors:** Artur Kubilis, Ali Abdulkarim, Ahmed M. Eissa, Neil R. Cameron

**Affiliations:** 1 Department of Chemistry, University of Durham, South Road, Durham, DH1 3LE, UK

## Abstract

Despite the low complexity of their components, several simple physical systems, including microspheres, coacervate droplets and phospholipid membrane structures (liposomes), have been suggested as protocell models. These, however, lack key cellular characteristics, such as the ability to replicate or to dock with extracellular species. Here, we report a simple method for the *de novo* creation of synthetic cell mimics in the form of giant polymeric vesicles (polymersomes), which are capable of behavior approaching that of living cells. These polymersomes form by self-assembly, under electroformation conditions, of amphiphilic, glycosylated block copolymers in aqueous solution. The glycosylated exterior of the resulting polymeric giant unilamellar vesicles (GUVs) allows their selective interaction with carbohydrate-binding receptor-functionalized particles, in a manner reminiscent of the cell-surface docking of virus particles. We believe that this is the first example of a simple protocell model displaying cell-like behavior through a native receptor-ligand interaction.

Over the last decades, the structural and molecular basis of cellular function has been elucidated. Cells are complex, hierarchical entities, which perform a number of functions that include nutrient transport and secretion, evolution and differentiation, replication and division, as well as adhesion and arrest. How life arose from its prebiotic origins is still unknown, and possibly will not ever be elucidated^1^. The scientific community seeks synthetic routes to species displaying cell mimicry and function. A few systems have recently been proposed as synthetic species displaying cellular behavior^2^,^3^. Nevertheless, we are still far from a comprehensive synthetic model. Cellular structures that embody the minimal and sufficient complexity to still be capable of exhibiting one or more features of biological cells are termed as protocells or minimal artificial cells^4^,^5^. As early as the 1960s, the concept of artificial cell microencapsulation was first introduced by Chang and co-workers^6^. Biologically active materials including live bacteria, proteins, DNA and drugs were encapsulated in a semipermeable membrane, primarily, a polymeric membrane that provides protection for the enclosed materials from the harsh external environment. The encapsulation membrane allows for the metabolism of solutes and bi-directional exchange of nutrients and waste. In recognition of the fact that Nature uses a more complex molecularly-structured approach, alternative protocell models are proposed which are based on supramolecular assemblies^7^,^8^. Self-assembled lipid vesicles (liposomes) are often chosen for minimal cell mimics due to the resemblance of their phospholipidic bilayer membrane to that of biological cells^9^. Polymer vesicles (polymersomes) are alternative cell mimicking structures of higher stability and with tunable membrane rigidity and permeability^10^, compared to liposomes. Furthermore, they can present biologically active functionalities on their external surface by self-assembly of suitably functionalized amphiphilic block copolymers^11^,^12^.

Recent developments in the field of cell biomimicry have made it possible to design advanced cellular structures^13^. Compartmentalized vesicles (vesicles-in-vesicles) have been established where each compartment can be independently made and loaded with different active materials; mimicking organelles in cells^14^. In addition, vesicles with a gelified interior (as a cytoplasm mimic) that can provide better stability and shape integrity have been developed. Marguet *et al*.^15^ combined both concepts of compartmentalization and a gel cavity in vesicles to achieve a more structurally advanced cell model.

The second rational step towards cell biomimicry is to introduce some “living” functional aspects (such as metabolism, replication or adaptability) to the existing cellular structural models. One such aspect is cellular internalization, in which cells take up a variety of external species including macromolecules, nanoparticles (e.g. viruses) and bacteria. Internalization occurs by various mechanisms, including endocytosis, the key stage in which is the docking of an external species to the cell membrane, followed by an invagination of the fluid bilayer and complete wrapping of the species in question and ultimately its transportation to the intracellular milieu encapsulated within a vesicle^16^,^17^. A sub-set of different endocytosis mechanisms is initiated by specific ligand-receptor interactions^18^. These receptor-mediated endocytosis (RME) processes are used by the cell to internalize a variety of nutrients, hormones, growth factors and other macromolecules, and are exploited by viruses as a means to gain entry into the cell^19^.

Carbohydrates are commonly encountered ligands for cell surface receptor proteins (lectins) and, indeed, many biological processes in mammalian cells, such as initiation of the inflammatory cascade, virus docking, fertilization and cancer cell metastasis, are mediated by carbohydrate-lectin interactions^20^,^21^. In many cases, carbohydrate-lectin binding leads to RME and internalization of the sugar-bearing cargo. Sugar-lectin binding typically displays high specificity despite the fact that interactions between individual sugars and lectins are unusually weak (K_a_ ca. 10^3^ M^−1^)^22^. This high specificity occurs through the ‘cluster glycoside’ effect, whereby many copies of the same sugar are presented to the lectin, leading to much higher K_a_ values (10^9^–10^12^ M^−1^)^23^. Consequently, multivalent glycosylated macromolecules, such as dendrimers (glycodendrimers) and linear polymers (glycopolymers), bearing many copies of the same sugar^24^, have been demonstrated to give binding to lectins that is massively enhanced compared to the individual sugar^23^,^25^.

At present, no structural cell mimics that can interact specifically with extracellular species in solution via receptor-ligand binding have been reported. Successful internalization of nanoparticles into liposomes^26^ and polymersomes^27^ has been shown recently as an attempt to mimic the phagocytosis process of living cells. However, in both cases, an external stimulus, such as a large concentration gradient^27^ or an optical trap^26^, was required to induce the uptake process. Here, we present the spontaneous and selective interaction between stable and robust cell-sized polymersomes, which have sugar moieties presented on their surface, and lectin-functionalized particles. The polymersomes are formed by self-assembly of amphiphilic glycopolymers, which were prepared using the RAFT^28^ polymerization technique.

## Results and Discussion

**Figure 1:**
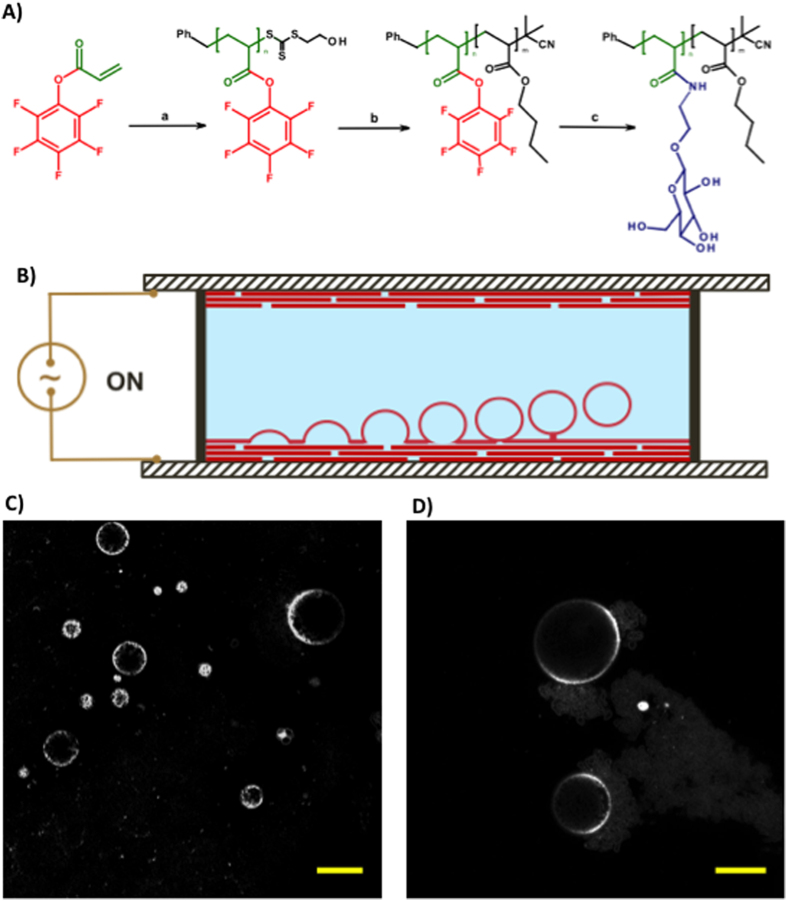
Preparation of glycosylated giant unilamellar vesicles (glyco-GUVs) from amphiphilic glycopolymers. (**A**) Synthesis of amphiphilic glycopolymers by (a) RAFT polymerization of pentafluorophenyl acrylate (BHECTT and AIBN, benzene, 70 °C, 6 h); (b) chain extension with *n*-butyl acrylate (*n*-butyl acrylate, AIBN, benzene, 70 °C, 6 h followed by excess AIBN, toluene, 80 °C, 3 h); displacement of pentafluorophenol by β-D-glucosyloxyethylamine (TEA, DMF–water 50:50, ambient temperature). (**B**) Schematic of electroformation apparatus for the construction of GUVs. A polymer film is deposited onto ITO-coated glass slides, which are separated by a rubber O-ring. The chamber is filled with sucrose solution and a sinusoidal electric field is applied. GUVs form by budding off from the film on the conductive substrate. (**C,D**) Fluorescence microscopy images of glycosylated GUVs stained with rhodamine B octadecyl ester perchlorate (scale bar is 20 μm).

We first utilized RAFT to polymerize an activated ester monomer, pentafluorophenyl acrylate (PFPA), followed by chain extension with n-butyl acrylate (n-BA) to produce a reactive block copolymer precursor for subsequent modification with amine-functionalized sugars (Fig. 1A). PFPA was first polymerized using benzyl 2-hydroxyethyl carbonotrithioate (BHECTT) as a chain transfer agent (CTA) (Table S1). The P(PFPA) as macroRAFT agents were used to polymerize n-BA to produce block copolymers with different compositions. After purification by reprecipitation, the block copolymers were analyzed by SEC which showed a monomodal distribution with dispersities of ca. 1.2 (Table S2). Prior to coupling with aminoethyl glucoside, the CTA end group was removed by treatment with AIBN. Under optimized experimental conditions, high yields with total consumption of pentafluorophenyl ester as revealed by ^19^F-NMR spectroscopy, were achieved. Further evidence of successful attachment of the sugar moieties was provided by FTIR spectroscopy (see SI).

Giant vesicles were prepared by self-assembly of the amphiphilic p(NβGluEAM-*b*-BA) glycopolymers using the electro-formation method (Fig. 1B), which has been shown to be efficient for producing giant unilamellar vesicles (GUVs) in high yields with narrow size distribution and few defect structures^29^,^30^. An AC field was applied across a conducting substrate onto which the glycopolymer was coated, causing vesicles to bud off from the surface. Application of optimized electro-formation conditions on one of the synthesized glycopolymers, namely p(NβGluEAM_5_-*b*-BA_50_), led to the formation of stable glycosylated GUVs (glyco-GUVs) with high yields (77 ± 8 vesicles per square mm) and average diameter of 20.0 ± 2.0 μm (Fig. 1C,D).

In order to utilize these glyco-GUVs as cell mimics, we needed to understand their response to changeable environmental conditions and permeability to various substances. We found that the glyco-GUVs responded to changing osmotic pressure; hypertonic conditions trigger shrinking of the vesicles while hypotonic conditions induce swelling. The glyco-GUVs are approximately 2.5 times more susceptible to negative osmotic pressure than positive. The average vesicle diameter decreases linearly by 19.7 ± 2.0% with an increase of negative osmotic pressure to −24.4 atm; however an increase in negative osmotic pressure beyond this value does not induce further changes in the average diameter of vesicles. Vesicles are able to withstand a negative osmotic shock higher than −24.4 atm and adapt to the altered osmolality; however, upon applying an osmotic shock lower than −24.4 atm the majority of the glyco-GUV population collapses and the remainder adjusts their average diameter to reduce the osmotic gradient.

Before employing these glyco-GUVs in interaction studies with receptor (lectin) – functionalized particles, it was necessary to demonstrate the availability of the pendent glucose moieties present on the vesicles’ surface for lectin binding. A turbidity assay was performed whereby 240 μl of a GUV solution was added to 600 μl of a Concanavalin A (Con A) solution in HEPES buffer (2 mg/mL). A steady increase in A_450nm_ was observed over 60 minutes caused by increasing sample turbidity (Figure S9). This is caused by agglomeration of glyco-GUVs, which present a multivalent display of glucose units to Con A which is itself multivalent (a tetramer at pH = 7.4).

**Figure 2:**
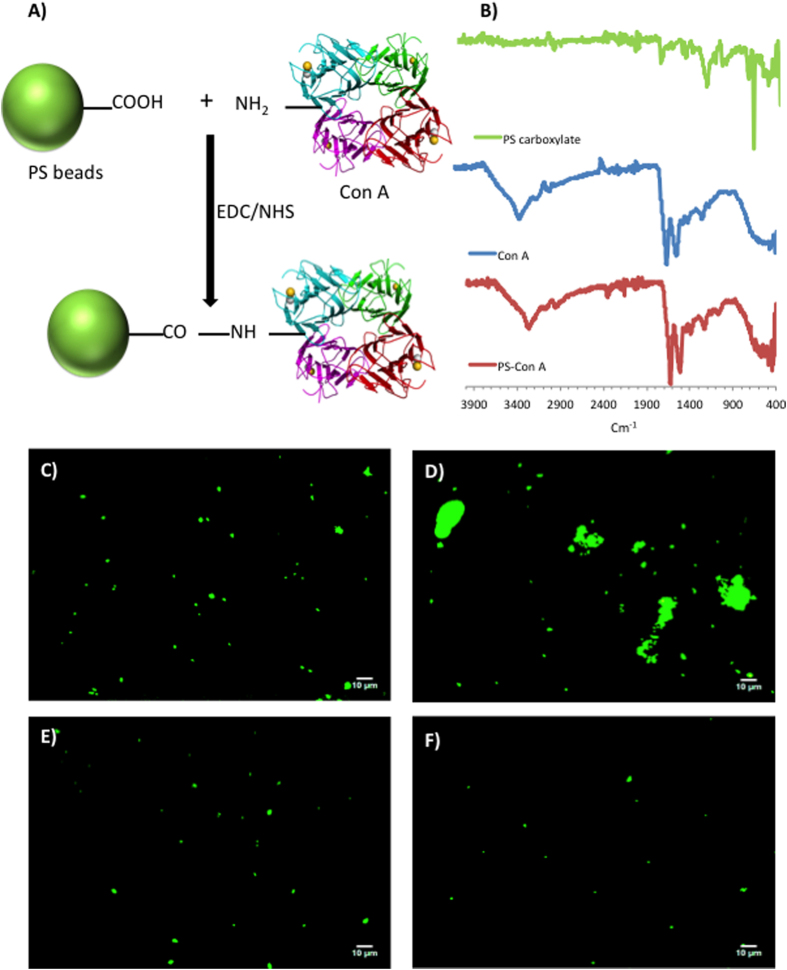
Preparation of lectin-functionalized fluorescently labelled polystyrene beads and their ability to bind multivalent glucosyl polymers. (**A**) Concanavalin A (Con A) was immobilized onto FITC-polystyrene beads (d = 1μm) possessing surface carboxylic acid groups (FITC-PS-CO_2_H) by EDC/NHS coupling. (**B**) ATR-FTIR spectra of (from top): FITC-PS-CO_2_H beads before reaction with Con A; powdered Con A lectin; FITC-PS-CO_2_H beads after reaction with Con A. (**C,D**) Fluorescence micrographs of suspensions of Con A – functionalized FITC-PS-CO_2_H beads in HEPES buffer (**C**) before and (**D**) after addition of a water-soluble multivalent glucosyl polymer. (**E**) Con A functionalized FITC-PS-CO_2_H beads in HEPES buffer after addition of a water-soluble multivalent fucosyl polymer (fucose does not bind to Con A). (**F**) unreacted FITC-PS-CO_2_H beads in HEPES buffer after addition of a water-soluble multivalent glucosyl polymer.

Con A–functionalized polystyrene (PS) beads were prepared as model extracellular receptor functionalized species to study their binding interactions with our glyco-GUVs (Fig. 2A,B). Commercially available carboxylate-modified PS beads were conjugated with Con A using carbodiimide coupling chemistry. Con A has a strong affinity for glucose–containing glyco–conjugates^31^. In order to probe the specificity of interactions between Con A–functionalized PS beads and glycopolymers, we conducted a microscopic assay whereby we added an aqueous solution of glucose– or fucose–containing multivalent glycopolymers to a suspension of Con A–functionalized PS beads in HEPES buffer (fucose has no binding affinity for Con A). On addition of the glucosidic polymer, the lectin–functionalized PS beads were seen to agglomerate very rapidly; conversely, on addition of the fucosidic polymer, no change in the agglomerated status of the beads was apparent (Fig. 2C–F). This agglomeration is due to specific binding interactions between the glucoside and subsequently potential crosslinking. The experiment was repeated using the carboxylate-modified PS beads, whereupon no agglomeration occurred, confirming that binding is caused specifically by carbohydrate–lectin interactions (Fig. 2C–F).

We next studied the interaction between our glyco-GUVs and Con A–functionalized PS beads as model extracellular objects. Confocal microscopy was used to visualize the interactions. In order to eliminate any potential errors and misinterpretations of data produced by non – lectin mediated interactions, two types of control experiments were performed: glyco-GUVs incubated with unfunctionalized PS beads (the original carboxylate-modified PS beads); and glyco-GUVs incubated with RCA_120_ – functionalized PS beads (RCA_120_ has no affinity to β-linked glucose moieties). All experiments were replicated in triplicate with an incubation time of 18 h, to allow significant numbers of interactions between beads and GUVs to occur. Upon overnight incubation of the glyco-GUVs with the unfunctionalized PS beads, very few examples of a bead next to a GUV were observed; however, the majority of the beads were distributed randomly and remained at the bottom of the visualization chamber. The percentage of interaction between the glyco-GUVs and the unfunctionalized beads, defined as the percentage of glyco-GUVs with an adjacent bead, did not exceed 6.5% in each of the observed samples. Similarly, upon overnight incubation of the glyco-GUVs with the RCA_120_–functionalized PS beads, a small number of interactions between the two species were observed; however the majority of RCA_120_–functionalized PS beads were dispersed randomly in the sample. The percentage of interaction between the glyco-GUVs and the RCA_120_–functionalized PS beads varied from 6 to 9%, which is slightly higher than that determined for the unfunctionalized PS beads.

**Figure 3:**
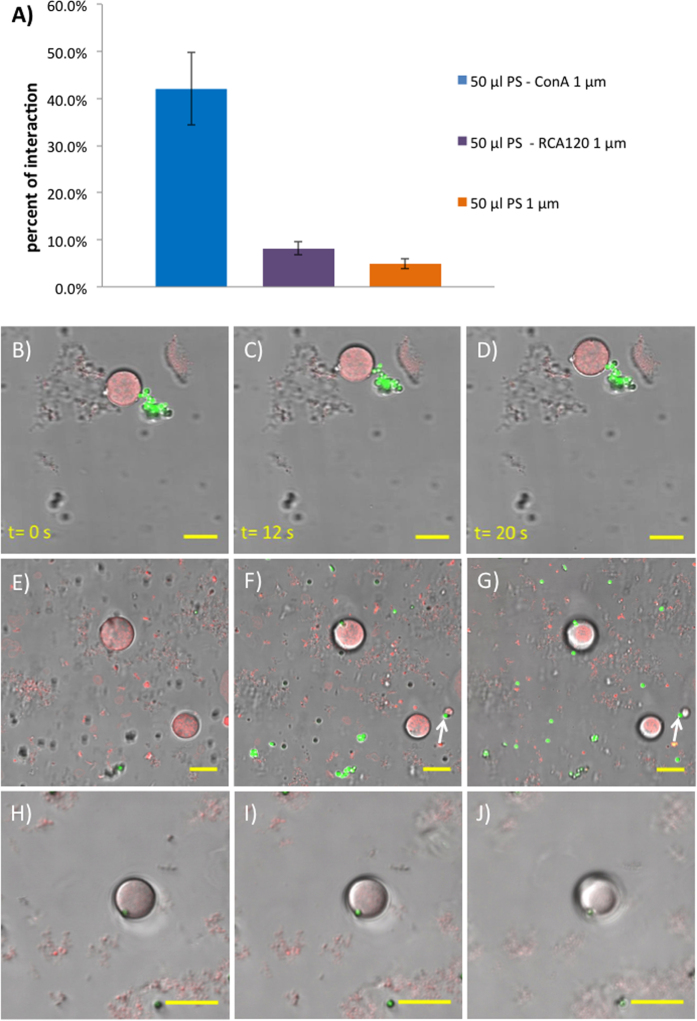
Glycosylated GUVs interact with lectin-functionalized PS beads through specific, multivalent sugar-lectin binding. (**A**) Bar chart showing frequency of beads interacting with glyco-GUVs, from left to right: FITC-PS-Con A; FITC-PS-RCA_120_; FITC-PS-CO_2_H (RCA_120_ is a β-galactosyl specific lectin). (**B–D**) Time-lapse confocal microscopy images showing a cluster of FITC-PS-Con A beads (green) bound strongly to a glyco-GUV (red). Both the beads and the GUV move in concert. (**E**–**J**) Z-stack confocal microscopy images showing (arrows) two examples of FITC-PS-Con A beads (green) bound to the surface of glyco-GUVs (red). Inter-focal plane distances: (**E,F**) 3.91 μm; (**F**,**G**) 1.87 μm; (**G,H**) 2.10 μm; and (**H,I**) 3.57 μm. (**B–I**) are still images from videos, full versions of which are available in SI.

Following these control experiments, we incubated our glyco-GUVs with the Con A–functionalized PS beads under the same conditions used for the control experiments. We observed in this case many examples whereby a bead appeared to attach to the surface of a glyco-GUV. Repeat experiments (n = 4) gave consistent results. Based on the collected data, the average percent of interaction between the glyco-GUVs and the Con A – functionalized PS beads was determined to be 42.0 ± 7.8% which is approximately five times higher than those with the RCA_120_ – functionalized PS (8.2 ± 1.4%) and eight times higher than those with the unfunctionalized PS beads (4.9 ± 1.0%) (Fig. 3A).

The strength and stability of the ligand – receptor interactions was assessed by recording the behavior of the species over a period of time. Figure 3B–D shows a glyco-GUV that is attached to a group of beads *via* a single bead – GUV connection. We presume that bead aggregation is caused by some free glycosylated polymer chains or nanostructures (eg micelles) that are too small to be observed by confocal microscopy. Time-lapse images show that the beads and GUVs move in concert, demonstrating that the strength and stability of the sugar-lectin binding interaction is sufficient to withstand translation from Brownian motion. Furthermore, the precise location of beads relative to GUVs was investigated by microscopy. Successive confocal microscopy images at different focal planes (Z-stack images) indicated that beads located adjacent to GUVs were indeed interacting strongly with the vesicle membrane (Fig. 3E–J). As the focal plane is lowered from roughly mid-way through the large GUV in the centre of the image (Fig. 3E), the bead appears (Fig. 3F) then increases in intensity (Fig. 3G), indicating that the bead is located next to the lower half of the GUV. Also seen in these images is a smaller GUV interacting with a bead (Fig. 3F,G – lower right, arrow). Evidence of a bead becoming embedded in a GUV membrane is presented in Fig. 3H–J (in the video in the SI, the GUV attempts to engulf the bead). At the lowest focal plane, it appears that the bead is to some extent buried in the GUV membrane (Fig. 3J). It should be noted that GUV aggregation induced by lectin-coated beads is unlikely due to the restricted motion of the GUVs in the confocal visualisation chamber.

**Figure 4:**
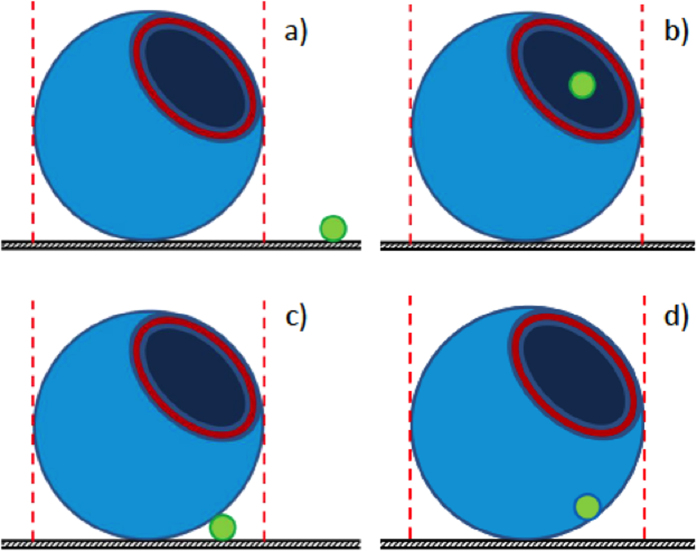
Schematic showing the possible different orientations of beads and glyco-GUVs. (**A**) bead and glyco-GUV are discrete from one another. (**B**) bead located inside the glyco-GUV. (**C**) bead interacting with the surface of the glyco-GUV. (**D**) bead embedded in the glyco-GUV membrane.

There are four possible locations of beads relative to GUVs (Fig. 4). GUVs have an internal aqueous pool consisting of a sucrose solution which causes them to sink to the bottom of the viewing chamber and so the GUVs rest on a substrate. We expect that confocal microscopy would easily reveal when beads are well-separated from GUVs (Fig. 4A). Beads internalized by GUVs (Fig. 4B) would be revealed by confocal microscopy at a focal plane mid-way through the GUV. An image in which the bead is clearly within the GUV membrane would be expected if internalization occurred. There is no clear evidence for such internalization in Fig. 3. A bead may be located adjacent to the GUV membrane whilst also resting on the substrate (Fig. 4C). We suspect that this is the situation described by Fig. 3E–G, where the fluorescence intensity of the bead is greatest at the lowest focal plane. The final possible orientation is when the bead is embedded in the GUV membrane, but not necessarily resting on the visualization chamber surface (Fig. 4D). Evidence for this relative orientation is provided in Fig. 3H–J. In particular, on lowering the focal plane it appears that the bead is interacting strongly with the GUV (Fig. 3J) and may indeed be buried in the GUV membrane.

In summary, we show that the outer membrane of giant polymersome protocells formed from glucose-bearing amphiphilic block copolymers are able to bind to microparticles that are decorated with the glucose-specific lectin Concanavalin A. Binding only occurs when both glucose and Con A are present on the surface of the polymersomes and microparticles, respectively. This behaviour mimics the binding of virus particles (e.g. influenza) to the surface of mammalian cells, which leads to viral particle entry and infection. This study, which we believe is the first to demonstrate receptor-mediated particle binding to giant polymersome protocells, may provide important insights for future research on protocells and minimal cell systems.

## Methods

PFPA was synthesized in a manner similar to that described in the literature^32^. Amphiphilic block glycopolymers of different molecular weights and compositions were synthesized by sequential RAFT polymerisation of PFPA and *n*-butyl acrylate, followed by transesterification of the PFP ester with 2′-aminoethyl-β-D-glucopyranoside and removal of the trithiocarbonate end group by treatment with AIBN. Polymers were characterized fully by NMR spectroscopy and SEC; in all cases, the obtained M_n_ agreed well with that predicted from the monomer to CTA ratio and dispersity values were in the range 1.1–1.2. Glyco-GUVs were prepared using an in-house fabricated electroformation apparatus consisting of two glycopolymer-coated indium tin oxide (ITO) glass slide electrodes, separated by a rubber O-ring spacer containing an aqueous sucrose solution, housed in PTFE and connected to an external AC power source. Lectins Con A or RCA_120_ were conjugated to commercially available FITC-labelled carboxylate-modified polystyrene latex beads by EDC/NHS coupling. Interactions between beads and glyco-GUVs were investigated by bright field and fluorescence confocal microscopy. The collected images were processed using ImageJ software. The Supplementary Information file gives full details of all synthetic procedures, characterization data for the polymers prepared, methods for GUV formation, as well as studies of GUV stability and their interaction with particles, including time-lapse videos showing GUVs interacting with particles.

## Additional Information

**How to cite this article**: Kubilis, A. *et al*. Giant Polymersome Protocells Dock with Virus Particle Mimics via Multivalent Glycan-Lectin Interactions. *Sci. Rep.* **6**, 32414; doi: 10.1038/srep32414 (2016).

## Supplementary Material

Supplementary Information

Supplementary video

Supplementary video

Supplementary video
