# Supplementary material for: Giant Polymersome Protocells Dock with Virus Particle Mimics via Multivalent Glycan-Lectin Interactions
Source: Sci Rep. 2016 Aug 31;6:32414. doi: 10.1038/srep32414 (PMC5006173; doi:10.1038/srep32414)
Supplement: Supplementary Information [file srep32414-s1.pdf]

SUPPLEMENTARY INFORMATION

Giant Polymersome Protocells Dock with Virus Particle Mimics via Multivalent Glycan-Lectin Interactions

A. Kubilis[a], A. Abdulkarim[a], A.M. Eissa[a,b]*, N.R. Cameron[a,b] *

[a] Department of Chemistry, University of Durham, South Road, Durham, DH1 3LE, U.K.

[b] Present addresses: Department of Materials Science and Engineering, Monash University, Clayton 3800, Victoria, Australia; School of Engineering, University of Warwick, Coventry, CV4 7AL, U.K.

* corresponding authors: neil.cameron@monash.edu; a.m.eissa@warwick.ac.uk

Table of Contents

Experimental **2**

Materials **2**

Instrumentation and Measurements **2**

Methods **3**

Synthesis of Pentafluorophenyl acrylate (PFPA) monomer 3

Synthesis of amphiphilic block glycocopolymers 4

Electro-Formation Procedure 10

Osmotic Shock Study 11

Turbidity Measurement 11

Preparation of lectin-conjugated PS beads 12

Interactions between GUVs and lectin-functionalized PS beads 12

References **17**

Experimental

Materials

Calcium (II) chloride (≥93.0 %), D-(+)-glucose (≥99.5 %), manganese (II) chloride (≥99 %), N - (2 - hydroxyethyl) piperazine - N′ - (2 - ethanesulfonic acid) (≥99.5 %), Nile Red (≥98.0 %; λ_{ex} 530 nm in methanol; λ_{em} 635 nm in methanol), Rhodamine B octadecyl ester perchlorate (≥98.0 %; λ_{ex} 554 nm in methanol; λ_{em} 575 nm in methanol), sodium chloride (≥99.5 %), α-D-glucopyranosyl β-D-fructofuranoside (≥99.5 %) were purchased from Sigma-Aldrich, UK. Benzyl 2-hydroxyethyl carbonotrithioate[1] and 2'-aminoethyl-β-D-glucopyranoside[2] were synthesized as described in the literature. Characterization data were in accord with published values. All other chemicals and reagents used in the synthesis were also purchased from Sigma–Aldrich and used without further purification.

HEPES buffer that was utilized in binding studies in order to maintain stability and activity of lectins presented on the surface of PS beads was prepared by dissolving N-(2-hydroxyethyl) piperazine-N′-(2-ethanesulfonic acid) (HEPES, 0.596 g, 0.01 M), sodium chloride (NaCl, 2.192 g, 0.150 M), calcium (II) chloride ($CaCl_2$, 0.055 g, 0.002 M) and manganese (II) chloride ($MnCl_2$, 0.063 g, 0.002 M) in 230 mL of ultrapure water in a volumetric flask. Upon full dissolution of the mixed chemicals, the required amount of ultrapure water was added to increase the total volume of buffer to 250 mL and the pH value was adjusted to 7.3 at ambient temperature. The prepared HEPES buffer was filtered using a syringe filter with pore size of 0.22 µm and stored at ambient temperature.

Instrumentation and Measurements

^{1}H-NMR spectra were recorded using the deuterated solvent lock on a Varian Mercury spectrometer at 400 MHz. Chemical shifts are quoted in ppm, relative to tetramethylsilane (TMS), as the internal reference. ^{13}C-NMR spectra were recorded at 101 MHz (2000 scans) using continuous broadband proton decoupling and a 3 s recycle delay and are therefore not quantitative; chemical shifts are quoted in ppm, relative to $CDCl_3$ (77.55 ppm).

FTIR spectra were recorded on a Perkin Elmer 1600 series FTIR spectrometer fitted with Diamond ATR crystal unit.

The electroformation apparatus was designed and manufactured in-house (Figure S1). The apparatus contains two copper slides which are connected to different electrical poles which operate as positive and negative electrodes. Two square indium tin oxide (In_2O_3/SnO_2, surface resistivity 8 - 12 Ω/sq) – coated glass slide electrodes were used, onto each of which a lipid or polymer solution was deposited to form a film. Using a rubber O-ring (10 mm), 150 µL of ultrapure water was sandwiched between the two ITO-coated slides. One glass slide was connected to the negative electrode and the other to the positive electrode. On applying an electric current, an electric field is created through the copper electrodes, glass slides, polymer film and water-based solution. The electric field initiates gentle film hydration and gradual detachment which facilitates vesicle formation.

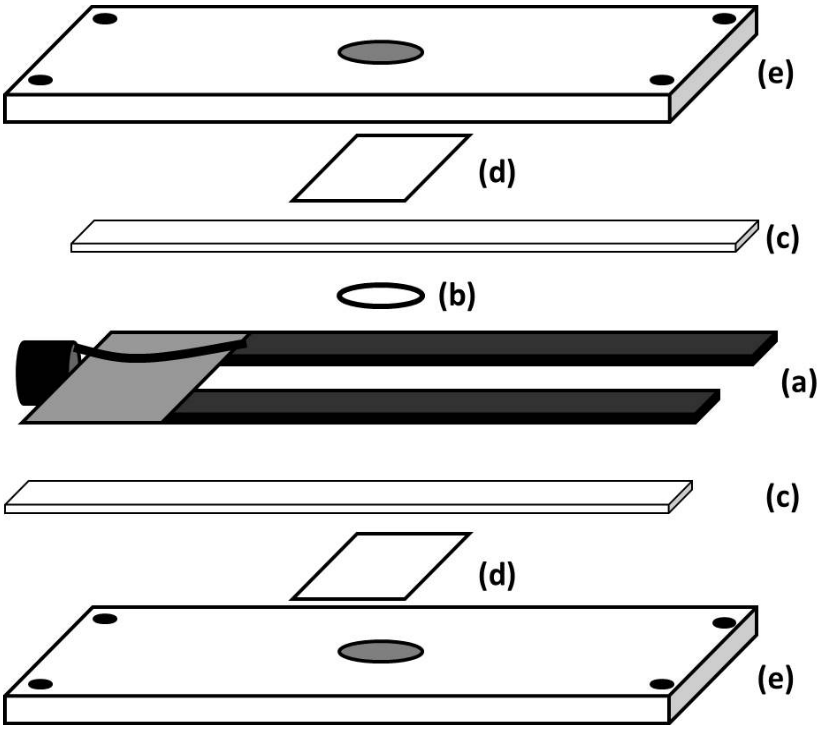

Figure S1. A sketch of the custom-made electroformation kit.

(a) Two Cu strips connected to electrical power supply; (b) rubber O-ring spacer; (c) PTFE isolating stripes; (d) indium tin oxide (ITO) coated glass slide electrodes; (e) PTFE electroformation kit housing

Confocal fluorescence microscopy observations were performed on a Nikon Diaphot microscope coupled with Bio-Rad Radiance confocal scanning system equipped with a Nikon Plain 503 NA 0.85 oil-immersion objective using He–Ne laser with excitation at 543 nm in combination with a PMT and emission filter at 570LP for detection.

Methods

Synthesis of Pentafluorophenyl acrylate (PFPA) monomer
PFPA was synthesized in a manner similar to that described in the literature.[3] Briefly, in a two-neck round-bottomed flask equipped with a stirrer bar, pentafluorophenol (PFP) (10 g, 0.0544 mol) and triethylamine (TEA) (6.65 g, 0.0658 mol) were dissolved in dry diethyl ether (100 mL). Acryloyl chloride (5.95 g, 0.0654 mol) was added dropwise using a syringe under cooling with an ice bath. After stirring for 18 h, the precipitated triethylamine hydrochloride salt was removed by filtration. The solvent was removed and the yellow crude product was purified using flash column chromatography (column material: silica gel; solvent: petroleum ether (40-60 °C)). The pure PFPA (colorless liquid) was stored at – 7 °C.

^{1}H NMR (400 MHz, $CDCl_3$): δ/ppm: 6.65 (d, 1H), 6.28 (dd, 1H), 6.13 (d, 1H). ^{13}C NMR (400 MHz, $CDCl_3$): δ/ppm: 161.65, 142.72, 141.21, 139.52, 137.91, 136.90, 135.42, 125.34, 124.99. ^{19}F NMR (400 MHz, $CDCl_3$): δ/ppm: -152.94 (d, 2F), -158.25 (t, 1F), -162.65 (d, 2F).

Synthesis of amphiphilic block glycocopolymers

1. RAFT polymerization of pentafluorophenyl acrylate (PFPA), block copolymerization with n-butyl acrylate (n-BA) and CTA-end group removal

PFPA (476 mg, 2 mmol), benzyl 2-hydroxyethyl carbonotrithioate (49 g, 0.2 mmol), AIBN (7 mg, 0.04 mmol) and benzene (4 mL) were placed into a Schlenk flask equipped with a stirrer bar. After degassing by purging with nitrogen under an ice bath for 30 min, the solution was heated to 70 °C and stirred for 6 h. Conversion of monomer to polymer was determined by ^{1}H NMR spectroscopy. The polymerization reaction was quenched by cooling and exposure to air. n-Butyl acrylate (2300 mg, 18 mmol) and AIBN (7 mg, 0.04 mmol) in benzene (6 mL) were added to the crude PFPA homopolymer solution. After degassing by purging with nitrogen under an ice bath for 30 min, the mixture was heated to 70 °C and stirred for 18 h. After quenching the reaction, the solvent was removed under reduced pressure and the product was reprecipitated twice from THF into cold methanol (at 0 °C). The block copolymer was dried under reduced pressure to yield a yellow powder which was then dissolved in toluene (30 mL) in the presence of a large excess of AIBN (30 eq.). After degassing by purging with nitrogen under an ice bath for 30 min, the mixture was heated to 70 °C and stirred for 6 h. After quenching the reaction, the solvent was removed under reduced pressure and the product was reprecipitated from THF into cold methanol (at 0 °C) to yield p($PFPA_{10}$-*b*-BA_{100}) as an off-white powder. The polymer was fully characterized by SEC, ^{1}H and ^{19}F NMR, and ATR-FTIR spectroscopies.

^{1}H NMR (400 MHz, DCM-d_2): δ/ppm: 7.13 (m, 5H), 3.95 (t, *J = 6.1 Hz*, 180H), 3.01 (bs, 10H), 2.42 (bs, 5H), 2.19 (bs, 180H), 2.06 (bs, 5H), 1.80 (m, 90H), 1.51 (m, 180H), 1.30 (m, 180H), 0.86 (m, 270H). ^{19}F NMR (400 MHz, DCM-d_2): δ/ppm: -153.29, -158.00, -163.18. ATR-FTIR: 1785 cm^{-1} (C=O PPFPA), 1730 cm^{-1} (C=O Pn-BA), 1520 cm^{-1} (C=C PPFPA), 795 cm^{-1} (C-F PPFPA). SEC (THF as eluent): M_n = 9,000 gmol^{-1}, $Đ$ =1.24.

Table S1: Characterization data for the obtained PFPA homopolymers.

Runs	[M]/[RAFT]/[I]	$M_{n,theor}$ g.mol^{-1}	$M_{n,SEC}$ g.mol^{-1}	*Đ*	$M_{n,NMR}$ g.mol^{-1}
p(PFPA)$_5$	5/1/0.2	1,435	2,360	1.17	2,150
p(PFPA)$_{10}$	10/1/0.2	2,625	3,050	1.15	3,100
p(PFPA)$_{25}$	25/1/0.2	6,194	8,190	1.26	8,100
p(PFPA)$_{50}$	50/1/0.2	12,144	15,700	1.27	15,240
p(PFPA)$_{75}$	75/1/0.2	18,094	16,300	1.26	24,040

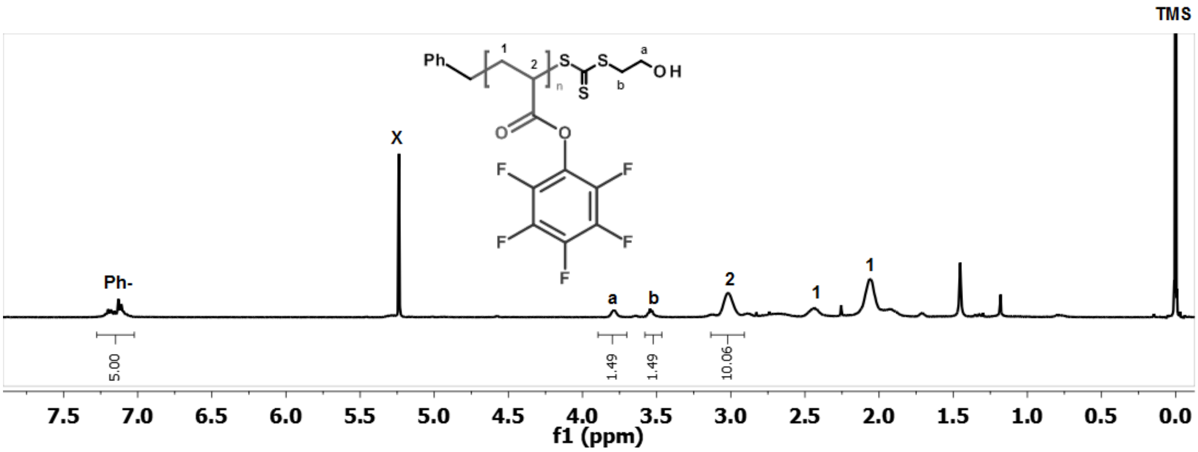

Figure S2. Representative ^{1}H-NMR spectrum of p(PFPA)$_{10}$ in DCM-d_2 (similar spectra were obtained for other PFPA polymers).

Table S2: Characterization data for the obtained PFPA/P(n-BA) block copolymers.

Polymer	[M]/[RAFT]/[I]	$M_{n,theor}$ g.mol^{-1}	$M_{n,SEC}$ g.mol^{-1}	*Đ*
p(PFPA$_{5}$-*b*-BA$_{100}$)	100/1/0.2	14,250	15,300	1.24
p(PFPA$_{10}$-*b*-BA$_{80}$)	80/1/0.2	12,880	17,000	1.10
p(PFPA$_{10}$-*b*-BA$_{100}$)	100/1/0.2	15,440	9,000	1.24
p(PFPA$_{10}$-*b*-BA$_{150}$)	150/1/0.2	21,850	30,470	1.12
p(PFPA$_{25}$-*b*-BA$_{250}$)	250/1/0.2	38,580	59,740	1.20

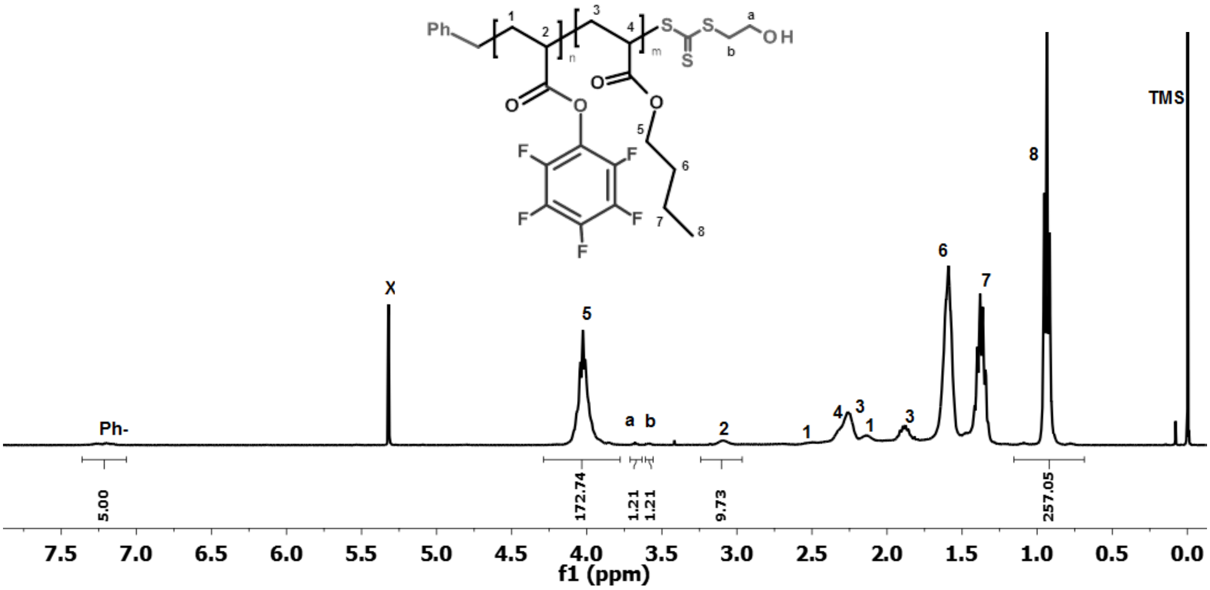

Figure S3. Representative ^{1}H-NMR spectrum of p(PFPA$_{10}$-*b*-BA$_{100}$) in DCM-d$_2$ (similar spectra were obtained for other PFPA polymers).

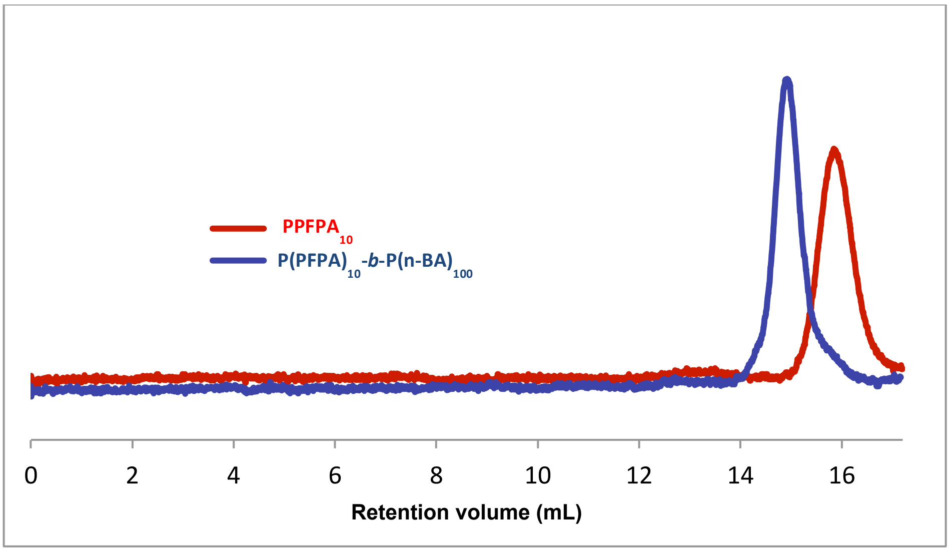

Figure S4. SEC traces of p(PFPA)$_{10}$ and p(PFPA$_{10}$-*b*-BA$_{100}$) (solvent peak was removed) (similar chromatograms were obtained for other homo- and block co-polymers).

2. Displacement of pentafluorophenyl groups with aminoglucoside

2'-aminoethyl-β-D-glucopyranoside (70 mg, 0.314 mmol) was mixed with triethylamine (70 µL) in water (1 mL). While stirring, the sugar solution was added slowly to p(PFPA$_{10}$-*b*-BA$_{100}$) (200 mg, 0.015 mmol) solution in DMF (2 mL). The mixture was stirred at 30 °C for 18 h. The product was then dialyzed against deionized water for 24 h and freeze-dried to yield p(NβGluEAM$_{10}$-*b*-BA$_{100}$) which was fully characterized SEC, ^{1}H and ^{19}F NMR, and ATR-FTIR.

^{1}H NMR (400 MHz, ($CDCl_3$ and CD_3OD; 1:1)): δ/ppm: 7.15 (b, 5H), 4.28 (b, 10H), 3.91 (b, 170H), 3.82 (b, 10H), 3.67 (b, 30H), 3.41 (b, 60H), 2.27 (b, 170H), 1.82 (b, 20H), 1.63 (b, 170H), 1.34 (b, 170H), 0.86 (b, 255H). ^{19}F NMR (400 MHz, ($CDCl_3$ and CD_3OD; 1:1)): δ/ppm: no signals. ATR-FTIR: 3340 cm^{-1} (-OH), 1730 cm^{-1} (C=O Pn-BA), 1650 cm^{-1} (C=O amide). SEC (DMF as an eluent, RI detector using PEO as a standard): M_n = 7,100 gmol^{-1}, $Đ$ =1.29.

Following the successful establishment of the stepwise synthesis and characterization of these amphiphilic glycopolymers, we developed a one-pot synthetic approach where it was not necessary to isolate the products for each step. Nevertheless, the final products were characterized fully. This was found to be a reliable approach which has dramatically reduced the time required for synthesis.

Table S3: Characterization data for the obtained amphiphilic gylycopolymers.

Polymer	$M_{n,theor}$ g.mol^{-1}	$M_{n,GPC}$ g.mol^{-1}	*Đ*
p(NβGluEAM$_5$-*b*-BA$_{10}$)	2,824	2,900	1.30
p(NβGluEAM$_5$-*b*-BA$_{30}$)	5,384	5,500	1.28
p(NβGluEAM$_5$-*b*-BA$_{50}$)	7,944	7,100	1.29
p(NβGluEAM$_{10}$-*b*-BA$_{100}$)	15,729	9,100	1.26
p(NβGluEAM$_{25}$-*b*-BA$_{250}$)	39,084	12,500	1.22

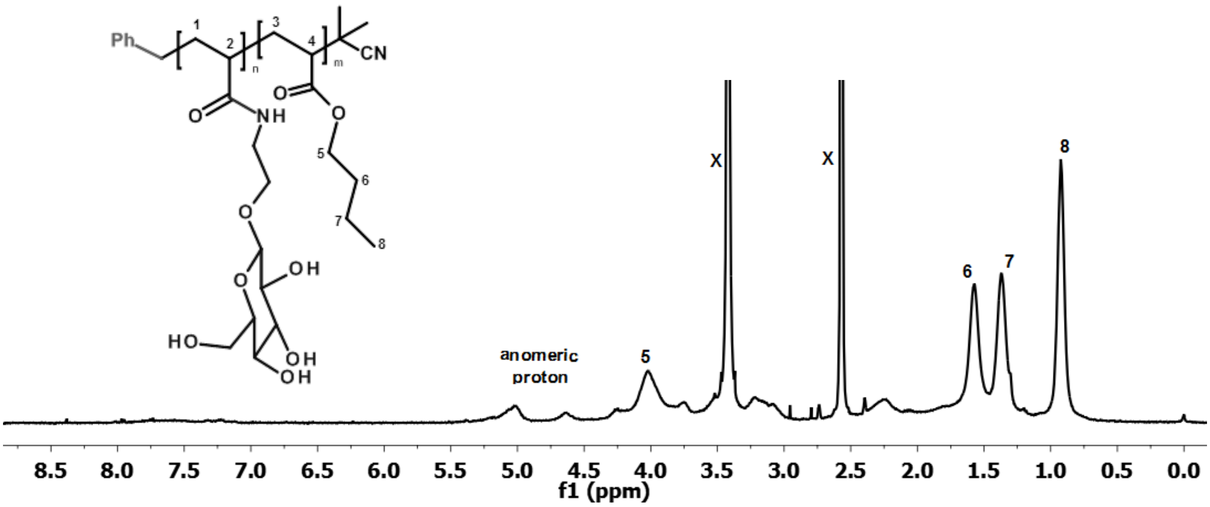

Figure S5. Representative ^{1}H-NMR spectrum of an amphiphilic glycopolymer in DMSO-d_6.

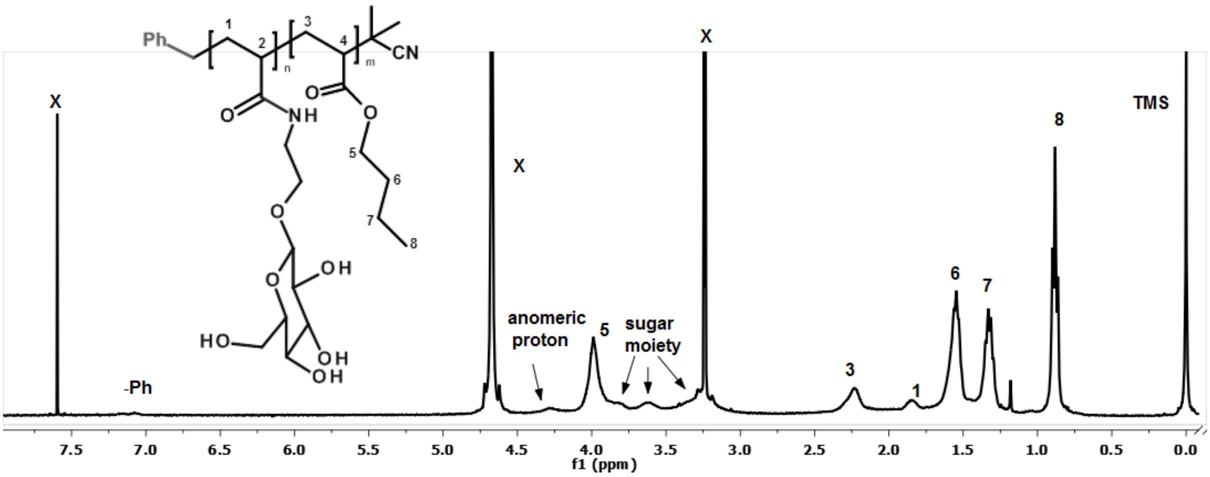

Figure S6. Representative ^{1}H-NMR spectrum of an amphiphilic glycopolymer in a mixture of $CDCl_3$ and CD_3OD.

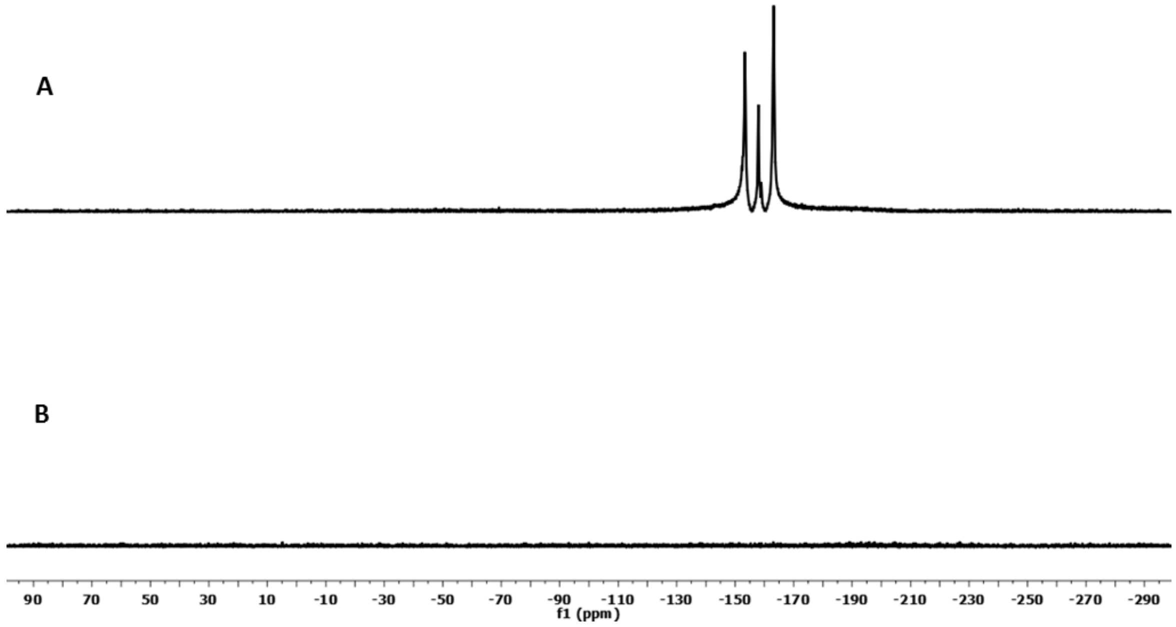

Figure S7. Representative comparison ^{19}F-NMR spectra of a p(PFPA-*b*-BA) block copolymer A) before and B) after treatment with aminoethyl-β-D-glucoside.

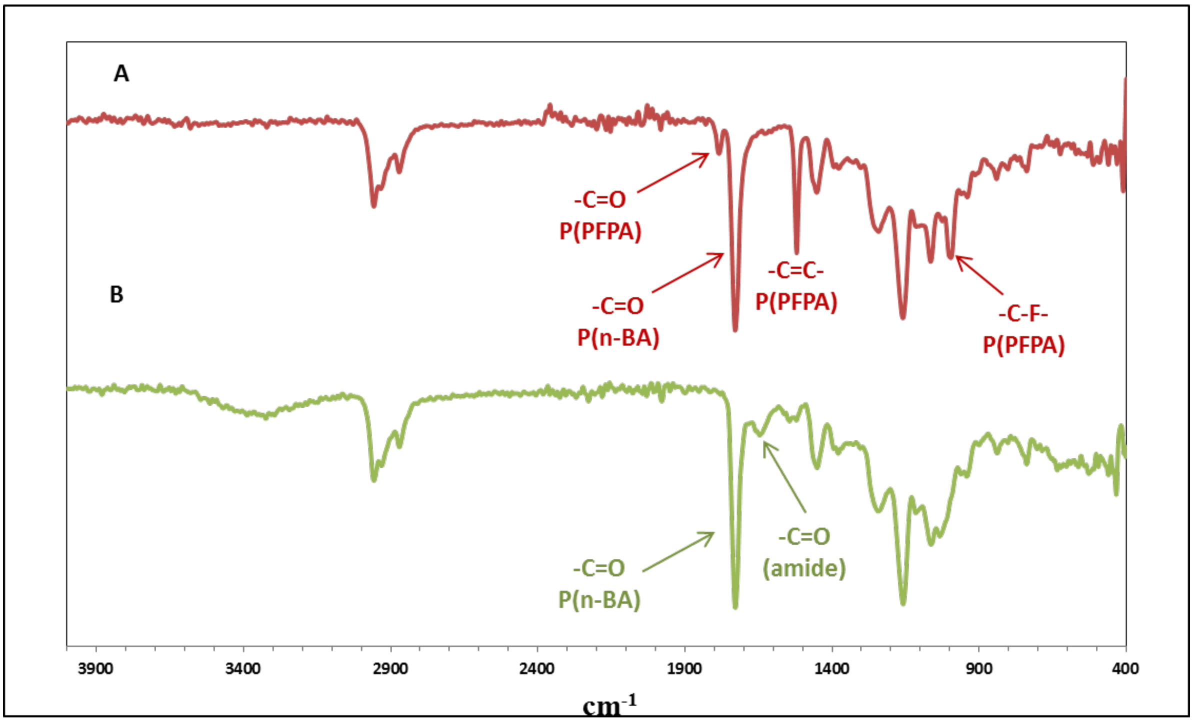

Figure S8. Representative comparison ATR-FTIR spectra of a p(PFPA-*b*-BA) block copolymer A) before treatment with aminoethyl glycosides and B) after treatment with aminoethyl glycosides

Electro-Formation Procedure

A 20 µL drop of p(NβGluEAM$_5$-*b*-BA$_{50}$) solution in THF/methanol (3:1) containing a lipophilic dye, Rhodamine B octadecyl ester perchlorate (0.001 mass %) was first deposited on each of two indium tin oxide (ITO) coated glass slide electrodes. The solvent was then evaporated under reduced pressure for 18h to form thin films on the ITO slides. Before applying an electric field of 9 V with frequency of 10 Hz and sinusoidal wave form, a rubber spacer was sandwiched between the two slide electrodes and filled with sucrose solution (100 mM) in ultrapure water. The experiment was run for 2h and then the sucrose solution containing the GUVs was carefully transferred to a visualization chamber with an isotonic sodium chloride to be ready for phase contrast observation under the bright field mode and confocal fluorescence microscopy.

The electro-formation method was utilized on all produced amphiphilic glycopolymers to test their GUV-forming ability. Upon reviewing results, it was found that p(NβGluEAM$_5$-*b*-BA$_{50}$) (see Table S3) had the greatest ability to form GUVs with narrowest size distribution and fewer defect structures. We therefore chose to only employ GUVs made from this amphiphilic glycopolymer in all experiments represented in this study.

Osmotic Shock Study

Hypertonic shock

Electroformation of p(NβGluEAM$_{10}$-*b*-BA$_{100}$) glycopolymer was performed in ultrapure water using the previously described procedure. 150 µl of the aqueous solution containing the GUVs was placed in a visualisation chamber filled with 600 µl of ultrapure water and the GUVs present were imaged. Hyperosmotic shock was performed by adding aqueous solution of NaCl (5 M) in six increments of 10 µL in order to increase the osmotic pressure. For each 10 µL addition, the sample was left for 2h to stabilise and then fully characterized.

Hypotonic shock

Electroformation of p(NβGluEAM$_{10}$-*b*-BA$_{100}$) glycopolymer was performed in 1 M sucrose solution using the previously described procedure. 150 µl of the sucrose solution containing the GUVs was placed in a visualisation chamber filled with 600 µl of 1 M sucrose solution and the GUVs present were imaged. Hypo-osmotic shock was performed by adding ultrapure water in three increments of 250 µL in order to decrease the osmotic pressure. For each 250 µL addition, the sample was left for 2h to stabilise and then fully characterized.

Turbidity Measurement

240 µL of GUV aqueous solution (approx. 0.53 mg/mL) was added to a cuvette containing 600 µl of Con A in HEPES buffer solution (2 mg/mL, approx. 18.9 µM assuming that Con A molar mass is 106 kDa). Absorbance of 450 nm light (A_{450nm}) was recorded using Variant Cary 100 Bio UV – Visible Spectrophotometer for the first 60 minutes every 5 minutes (Figure S2). Control experiments were performed using analogous protocol, utilising 2 mg/mL Con A solution in HEPES buffer and GUVs solution in HEPES buffer.

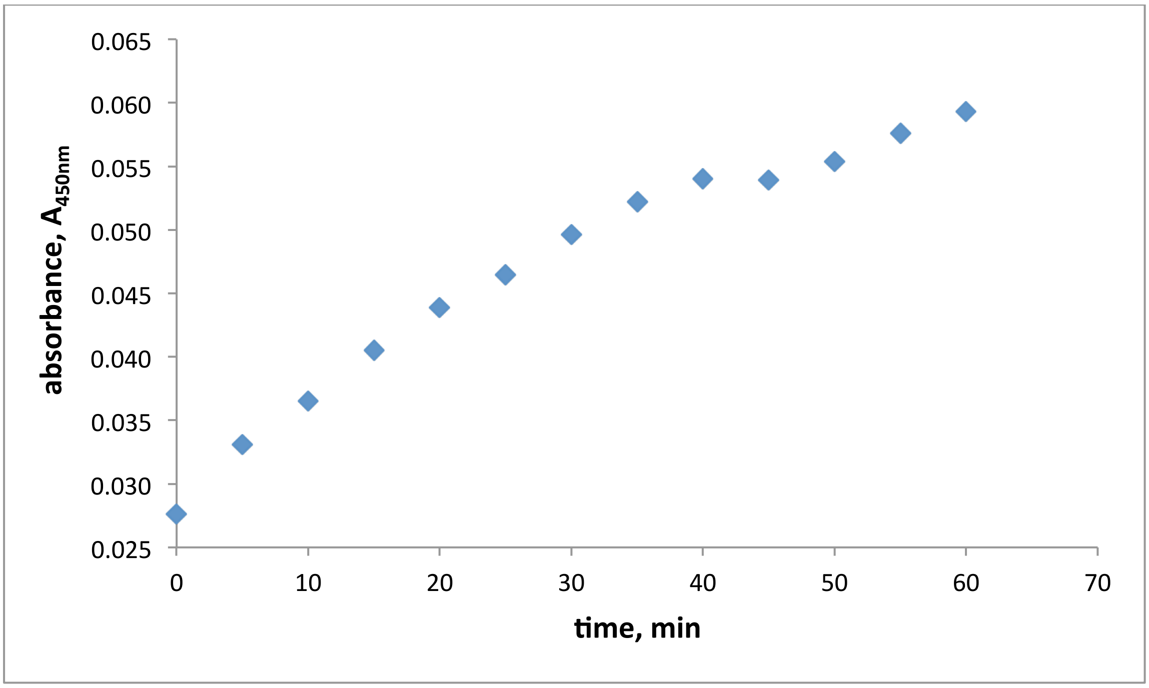

Figure S9. Assessment of the binding of glyco-GUVs of p(NβGluEAM$_{5}$-*b*-BA$_{50}$) to Con A by turbidimetry.

Preparation of lectin-conjugated PS beads

Commercially available FITC-labelled carboxylate-modified PS latex beads, mean size 1 µm, (500 µL, aqueous suspension, 2.5 %) were activated by stirring for 2h at ambient temperature in the presence of N-(3-Dimethylaminopropyl)-N′-ethylcarbodiimide hydrochloride (EDAC; 250 µL, 0.4 M) and *N*-hydroxysuccinimide (NHS; 250 µL, 2.8 M). Excess reagents were removed by dialysis (MWCO 3.5-5 kDa) against phosphate buffered saline (PBS; pH 7.4). The activated beads dispersion in PBS was added slowly into an Eppendorf tube containing Con A or RCA_{120} (5 mg) under continuous vortexing. The reaction was allowed to proceed for 18h. The excess coupling sites were blocked by incubating the beads dispersion with glycine solution (0.5 mL, 200 mg/mL) for 1h at ambient temperature. The beads dispersion was dialyzed (MWCO 12-14 kDa) against PBS and then bicarbonate-carbonate buffer (pH 9.5) to remove any unconjugated lectin. The buffer was replaced by ultrapure water. The lyophilized lectin-conjugated PS beads were stored at -20 °C. Prior to usage the functionalized beads were suspended in HEPES buffer at a required concentration.

Interactions between GUVs and lectin-functionalized PS beads

Following the preparation of GUVs and functional PS beads as described previously, 150 µL of GUVs in ultrapure water was added to a visualization chamber containing 600 µL of HEPES buffer solution and left for 30 min to allow stabilization of GUVs. After full characterization of the GUVs, 50 µL of PS beads (control experiments: unfunctionalized and RCA_{120}-functionalized PS beads; selective interaction studies: Con A-functionalized PS beads) was added to the GUVs solution. Following 15h of incubation, the samples were imaged using bright field and fluorescence confocal microscopy. Collected images were processed using ImageJ version 1.46r software (Figures S3-S5).

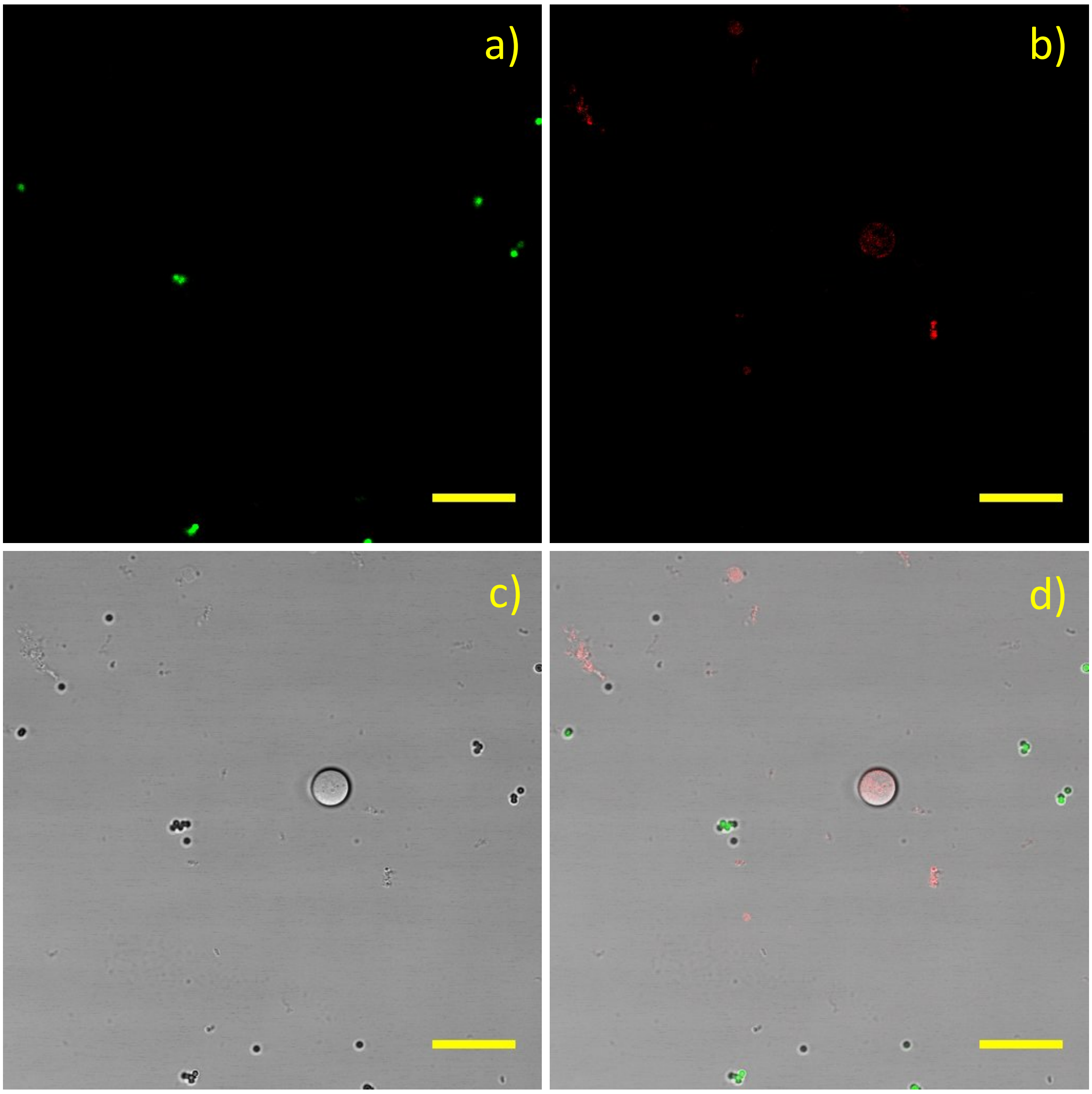

Figure S10. Microscopy images of interactions between unfunctionalized PS beads and glyco-GUVs: a) confocal green (Fluorescein 494 nm) channel showing the unfunctionalized PS beads; b) confocal red (Rhodamine B octadecyl ester perchlorate 554 nm) channel showing the glyco-GUVs; c) bright field channel; d) overlaid green, red and bright field channels. Scale bar size is 20 µm.

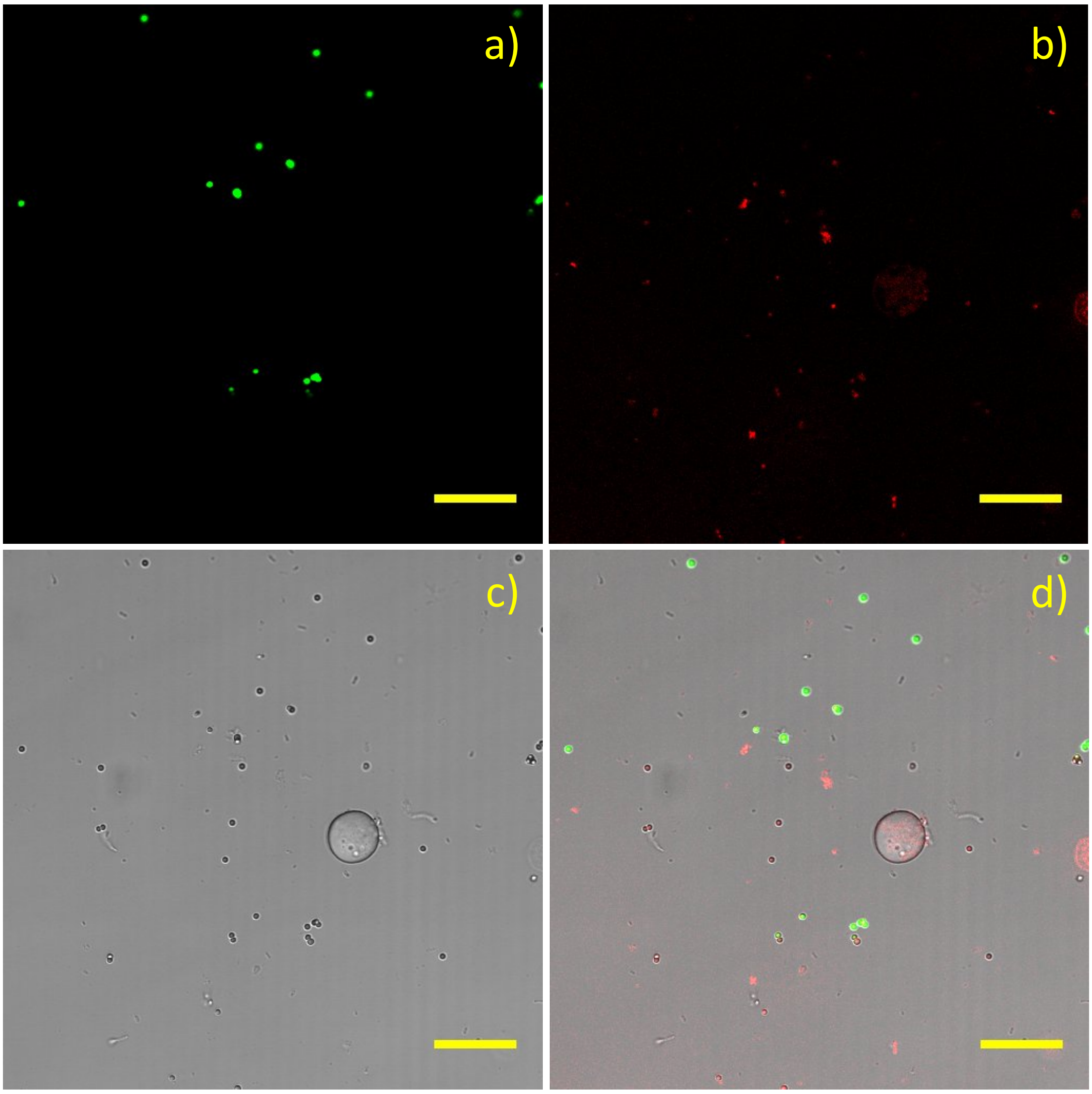

Figure S11. Microscopy images of interactions between RCA_{120} functionalized PS beads and glyco-GUVs: a) confocal green (Fluorescein 494 nm) channel showing the RCA_{120} functionalized PS beads; b) confocal red (Rhodamine B octadecyl ester perchlorate 554 nm) channel showing the glyco-GUVs; c) bright field channel; d) overlaid green, red and bright field channels. Scale bar size is 20 µm.

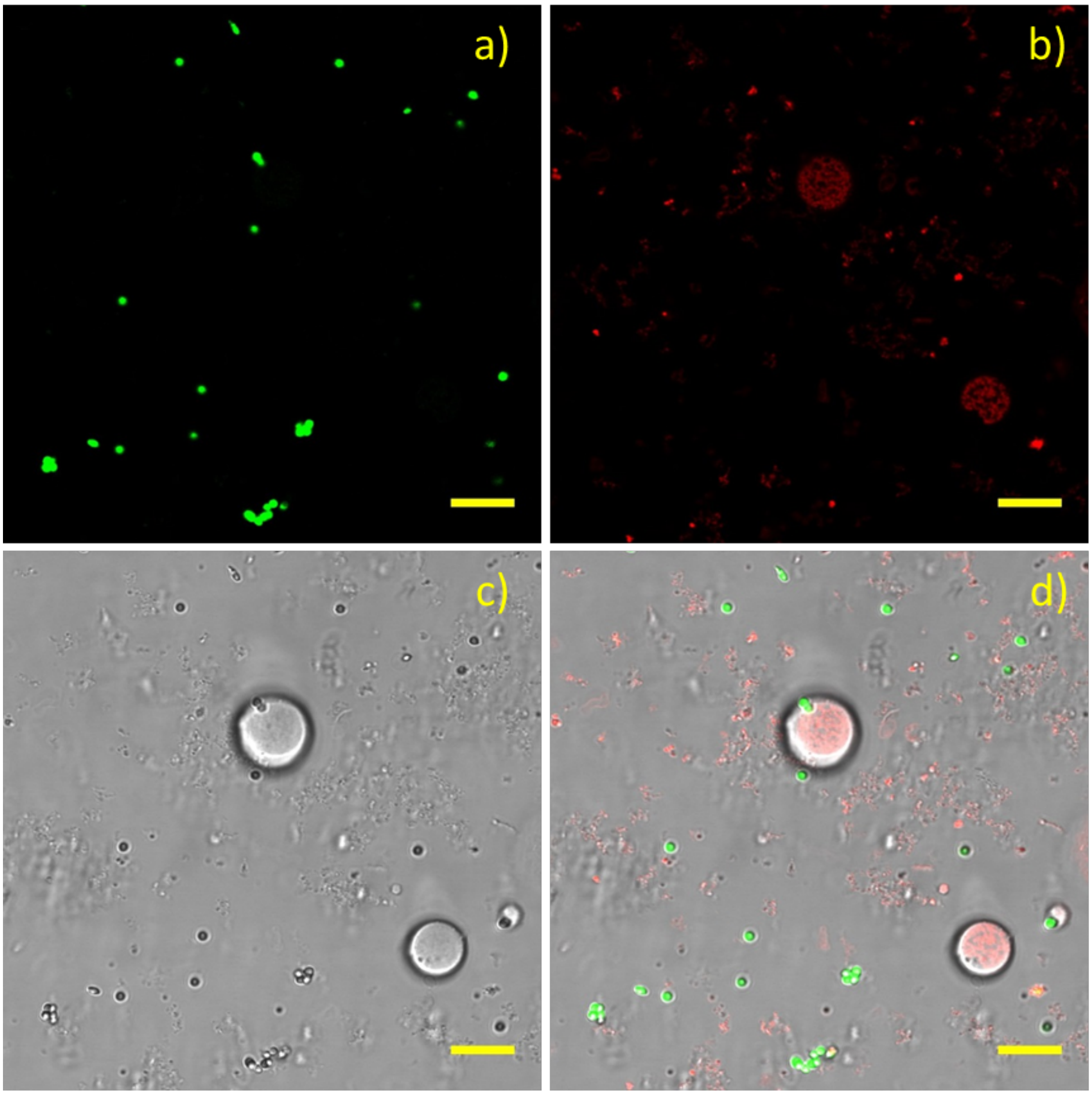

Figure S12. Microscopy images of interactions between Con A functionalized PS beads and glyco-GUVs: a) confocal green (Fluorescein 494 nm) channel showing the Con A functionalized PS beads; b) confocal red (Rhodamine B octadecyl ester perchlorate 554 nm) channel showing the glyco-GUVs; c) bright field channel; d) overlaid green, red and bright field channels. Scale bar size is 10 µm.

A)

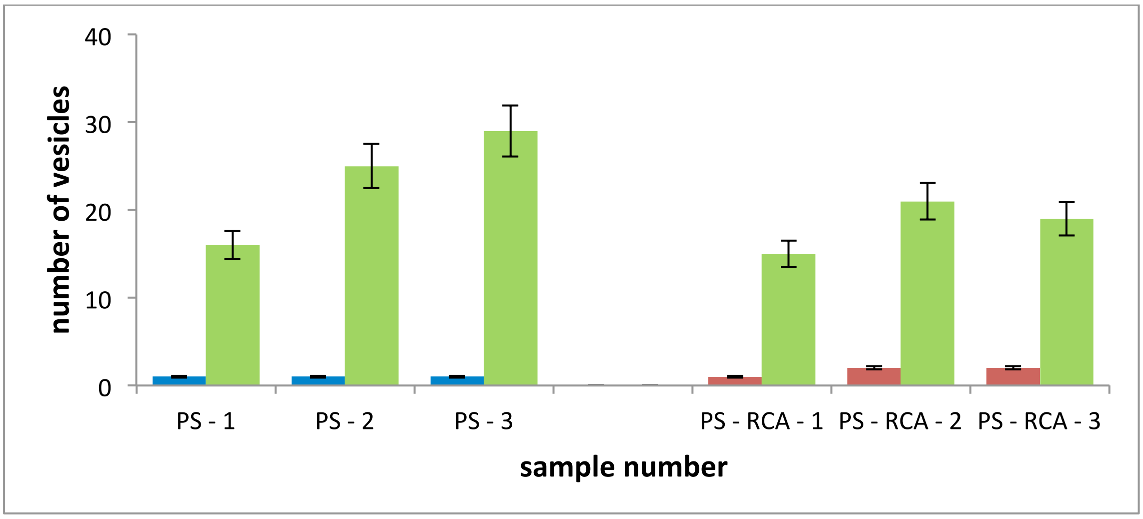

B)

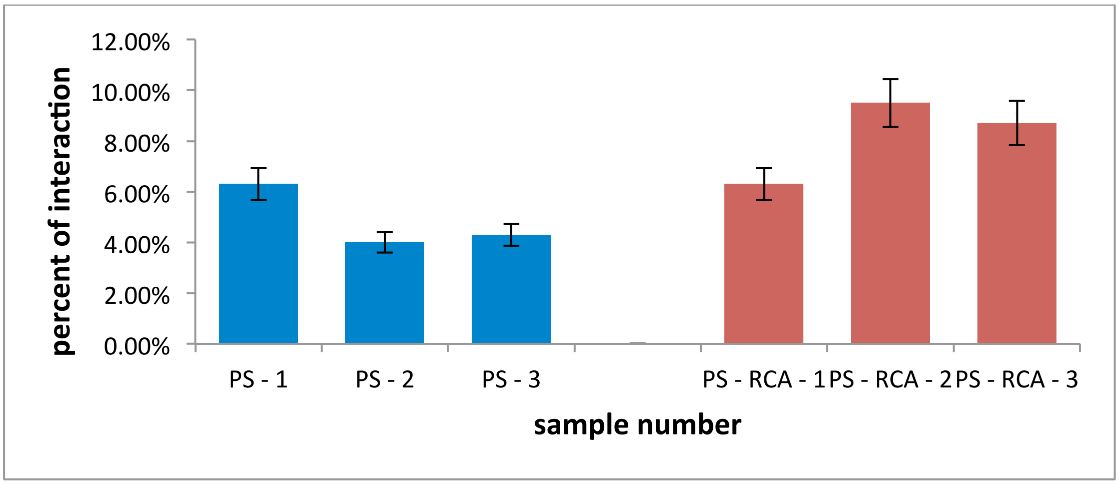

Figure S13. A) Number of glyco-GUVs interacting with unfunctionalized PS beads (blue bars) and RCA_{120} functionalized PS beads (red bars), and total number of glyco-GUVs observed in each sample (green bars); B) Percent of interaction of glyco-GUVs with unfunctionalized PS beads (blue bars) and RCA_{120} functionalized PS beads (red bars).

A)

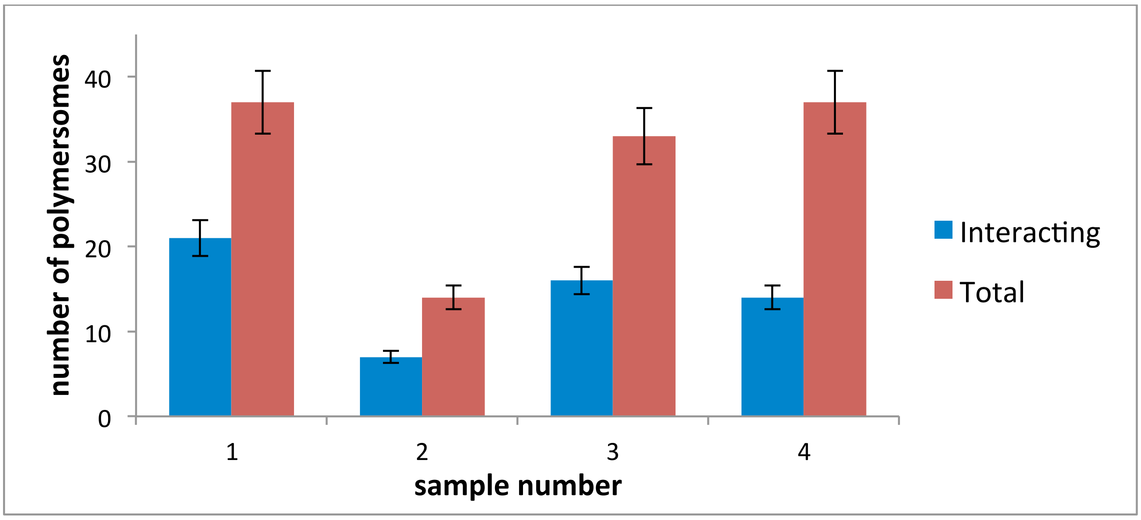

B)

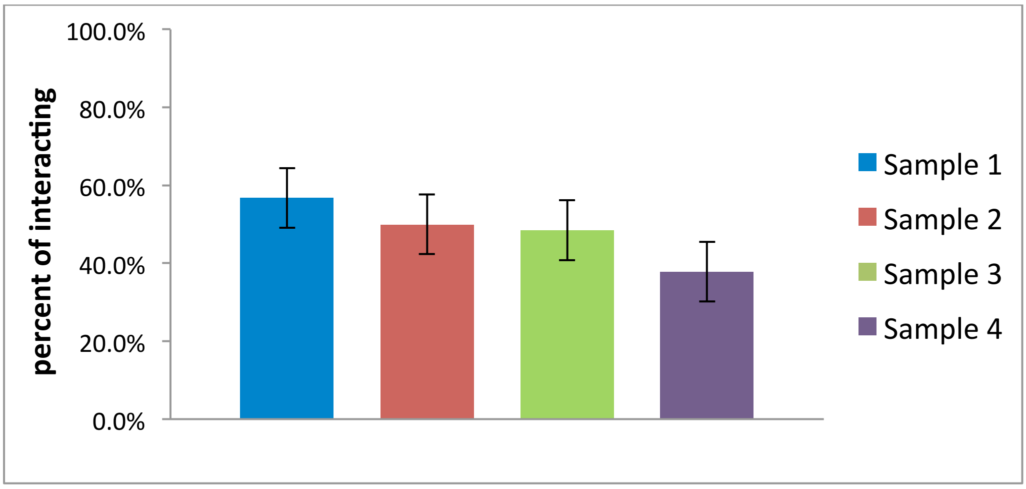

Figure S14. A) Number of glyco-GUVs interacting with Con A functionalized PS beads (blue bars) and total number of glyco-GUVs observed in each sample (red bars); B) Percent of interaction of glyco-GUVs with Con A functionalized PS beads.

Time-lapse videos showing glyco-GUVs interacting with Con A functionalized PS beads are also presented (Movie 1.mov; Movie 2.mov; Movie 3.mov).

References

1. Skey J, O'Reilly RK. Facile one pot synthesis of a range of reversible addition-fragmentation chain transfer (RAFT) agents. *Chem. Commun.* 2008(35)**:** 4183-4185.

2. Chernyak AY, Sharma GVM, Kononov LO, Krishna PR, Levinsky AB, Kochetkov NK*, et al.* 2-azidoethyl glycosides - glycosides potentially useful for the preparation of neoglycoconjugates. *Carbohyd. Res.* 1992, **223:** 303-309.

3. Jochum FD, Theato P. Temperature- and Light-Responsive Polyacrylamides Prepared by a Double Polymer Analogous Reaction of Activated Ester Polymers. *Macromolecules* 2009, **42**(16)**:** 5941-5945.
